# Management of Medicines Wastage, Returned Medicines and Safe Disposal in Malaysian Community Pharmacies: A Qualitative Study

**DOI:** 10.3389/fmed.2022.884482

**Published:** 2022-05-19

**Authors:** Kah Mun Chong, Kingston Rajiah, David Chong, Mari Kannan Maharajan

**Affiliations:** ^1^Student, Master in Pharmacy Practice, School of Postgraduate Studies, International Medical University, Kuala Lumpur, Malaysia; ^2^Gandhi Institute of Technology and Management (GITAM) School of Pharmacy, GITAM Deemed University, Hyderabad, India; ^3^Department of Pharmacy Practice, School of Pharmacy, International Medical University, Kuala Lumpur, Malaysia

**Keywords:** medicines wastage, unused medicines, expired medicines, medicines disposal, returned medicines

## Abstract

**Introduction:**

In supplying medicines to patients and consumers waste can occur in prescribing, dispensing, and leftover stages. Pharmacists in community pharmacies play a crucial role in dispensing and should share information on appropriate medicines disposal with consumers. This qualitative study explored how Malaysian community pharmacists manage medication wastage, returned medicines, and medicines disposal by eliciting their opinions on medicines wastage, the challenges faced, and feasibility of medicine return and safe medicine disposal in the setting of Malaysian community pharmacy.

**Methods:**

Telephonic interviews were conducted using a pre-validated interview guide among community pharmacists. Purposive sampling was used to ensure heterogeneity of participants in terms of gender, age, and position in the pharmacy. The interview was conducted until a point where no new information was obtained. Interview data were thematically analyzed.

**Results:**

The analysis identified nine themes organized into four domains. The results revealed that pharmacists have positive perceptions of the safe disposal of medicines. Pharmacists mentioned that medicine returns to service in community pharmacies are not common due to a lack of facilities in the management of unwanted, expired, and returned medicines. As such pharmacists have suggested a few ways to minimize medicinal wastage.

**Conclusions:**

Respondents aimed to minimize medicines wastage (unused medicines) in order to minimize loss of revenue. Respondents did not usually accept returned medicines due to the operational costs of safe disposal. Disposal of unused medicines was undertaken by centralizing the stocks at an organization facility before being disposed of by outsourced waste management companies.

## Introduction

Community pharmacies are the primary healthcare facilities most accessible to the public (1). The conventional roles of community pharmacists are mostly business orientated, where they focus more on prescription dispensing, selling over-the-counter drugs and running the pharmacy business (1, 2). Over time, the roles of community pharmacists have expanded to focus on quality and safe use of medicines in the primary healthcare system (2). Pharmacists involving in safe use of medication, including its storage, and disposal.

Considering the chemical and biological nature of medications, improper use and disposal of medications in community and hospital pharmacies led to economic losses and cause damage to the environment and society. This impact of medication wastage increased the awareness of potential waste minimization strategies to be implemented in community and hospital pharmacies. Medication waste can occur in the prescribing, dispensing, and leftover stages of the pharmaceutical supply chain (3). Pharmacists have the most influence while dispensing the “over-the-counter” medications. Here, one cause of medicine wastage is large manufacturers' packs which may contain a greater quantity of the medicine than that needed for treatment (4). The direct cost of medication waste is of major concern and can jeopardize the sustainability of healthcare systems (4). In the leftover stage, non-adherence is the most common cause of medicine wastage. This happens often among elderly patients, who may have difficulty in remembering to take medicines due to the large number of different products that they are required to take each day (5). For these patients, unused or forgotten medications are often left to expire and to be disposed of as garbage or into the sewage system (6).

Management of medication wastage, medicine return, and safe medicine disposal has been a global challenge in the sustainability of healthcare systems (7). Health policymakers, pharmaceutical organizations, healthcare professionals, and the wider community, emphasize this issue due to its undeniably detrimental effects on the environment, the economy, and patient safety (7, 8).

Pharmacists play an important role in minimizing medicine wastage and educating the public in safe medicine disposal (3, 9). Education on the appropriate disposal of medications is a key intervention for all pharmacy settings. In Malaysia, public hospital pharmacists could rely on the government's “Return Your Medicines” Program to encourage returns of unused and expired medications to minimize wastage of medicines and their inappropriate disposal (10). On the other hand, the practices of community pharmacists in the management of medication wastage, medicine returns, and safe medicine disposal have not been explored as there are no relevant governmental regulations or frameworks. The roles of community pharmacists in these practices could be enhanced and given greater emphasis, as community pharmacies are one of the most accessible health care facilities for medication-related advice. Therefore, this study aimed to explore the management of medication wastage, medicine return, and safe medicine disposal in Malaysian community pharmacies. It will be useful to understand the common practices and methods employed by community pharmacists in these activities, along with, their opinions concerning medicine wastage issues and the challenges faced, as well as the feasibility of medicine return and safe medicine disposal in Malaysian community pharmacies; this will facilitate concrete steps to be taken to encourage standard pharmaceutical waste management in community pharmacy settings.

### Ethical Considerations

The study was approved by the IMU Joint-Committee on Research and Ethics [MPP I-2020(07)].

## Materials and Methods

### Study Design

One-to-one telephone interviews were conducted using a pre-validated interview guide from December 2020 to June 2021. Community pharmacists were approached after their working hours, introduced to the purpose of the study, and verified for eligibility for the study; only eligible individuals were invited to participate. Eligible respondents were provided with an outline of the study objectives and informed of the time needed for the study. Only full-time, fully-registered pharmacists working in a community pharmacy were included, with the exclusion of locum community pharmacists and provisionally registered pharmacists. Participation was by voluntary written informed consent.

### Interview Guide

A semi-structured interview guide was created by reviewing the literature on medicine wastage, return of medicine, and safe disposal. The guide was modified as a result of suggestions and recommendations from experts in qualitative studies. Open-ended questions were preferred to provide interviewees with a full opportunity to convey their opinions and help in obtaining a greater understanding of issues. A pilot interview was conducted with two pharmacists. The questions were rephrased based on the pilot interview. The data collected during the pilot interview were not included in the results.

### Sampling Method and Sample Size

Purposive and snowball sampling was used to select participants to ensure heterogeneity of the participants' demographic characteristics in terms of their gender, age, and position in the pharmacy. Sampling was undertaken until the saturation point. The saturation point was determined by analyzing the collected data that indicated a further collection of data may not bring any new theme. In other words, the researchers reached a point where there were no new themes are generated from the data collected (11).

### Data Collection

Telephone interviews were conducted. The interview durations were between 30 to 45 min. All interviews were audio-recorded. The interview recordings were subsequently transcribed verbatim by a researcher. All transcripts were checked for accuracy by two researchers. The transcripts were subsequently returned to the respondents for comments and corrections. The final transcripts were stored in password-protected Microsoft Office Word document.

### Data Analysis

Transcribed data were coded and analyzed for emergent themes using thematic analysis, as per the approach and steps recommended by Braun and Clarke (12). The data were coded based on common themes, where the data were sorted into categories with similar trends. The codes were then labeled, and notes were written about the ideas which arise from them. The data were then grouped into themes and inductive thematic analysis was conducted as per the following steps. First, the researchers familiarized themselves with the data, then the initial codes were generated. Followed by suitable searching of themes, reviewing of themes, defining and naming themes were done. Finally, the report was produced (12). The data collection, coding, and interpretation were carried out in an iterative manner by a researcher and the resultant data were confirmed by two researchers.

### Data Trustworthiness

In this study, trustworthiness was established in several phases. To reduce researcher bias, a reflective journal was used while taking field notes. To address the data transferability, respondents' characteristics were mentioned. Completeness and credibility of the content were ensured by establishing the sample size based on saturation. To give proper meaning to the text, the unit of analysis was taken as sentences instead of letters or words.

## Results

Eighteen eligible community pharmacists participated in this study. Most of the respondents were aged 30 years or below. Ten of the respondents were female and eight of them were male. Most respondents had at least 5 years of experience working in urban areas as pharmacists; only one respondent was from a rural setting. Table 1 summarizes the demographic characteristics of the respondents.

**Table 1:** Demographic data of the respondents.

**Characteristics**	**Number of respondents**
**Age (Years)**
18–30	10
31–40	05
41 and above	03
**Gender**
Male	08
Female	10
**Experience in years**
Less than a year	05
1–5	09
More than 5	04
**Job description**
Pharmacist	16
Pharmacy manager	02
**Pharmacy location**
Urban	17
Rural	01

The thematic analysis identified nine themes organized into four domains: (1) Perception of pharmacists regarding medicine disposal (2) Availability of medicine return service in community pharmacies (3) Management of unwanted, expired, and returned medicines (4) Minimization of medicines wastage. Table 2 summarizes the themes and the domains.

**Table 2:** Identified domains and themes of the study.

**S. No**	**Domains**	**Themes and sub-themes**
1	Perception of pharmacists regarding medicine disposal	1. Safe medicine disposal practices
		2. Medicine Return Program
2	Availability of Medicine Returns Service in Community Pharmacies	3. Driving factors toward medicine return service
		3.1: Environmental impacts and pediatric poisoning
		3.2: Convenient location
		3.3: Promote government initiative
		3.4: Support from the company
		3.5: Incentives from the government
		4. Deterring factors toward medicine return service
		4.1: Lack of guidelines
		4.2: Operational costs and incineration fees
3	Management of unwanted, expired and returned medicines	5. Close-expiry medicines
		6. Expired medicine stocks and medicinal wastage
		7. Medicine returns from the public
4	Minimization of medicinal wastage	8. Pharmacy stocks
		9. Dispensed medications

### Domain 1: Perception of Pharmacists Regarding Medicine Disposal

#### Theme 1: Safe Medicine Disposal Practices

All the 18 respondents agreed that safe medicine disposal is beneficial for public health and acknowledged the negative effects of improper medicine disposal on the environment and healthcare costs.

“*We rarely see customers buying bulk-buying medications as they have to pay from their own pockets.” R5*

“*I think patients that are likely to have unused medications are those getting the medications from government hospitals, where the medications are dispensed at low prices or even free.” R12*

“*If people do not dispose of the medicine properly, it may affect the surroundings and cause hazards.”R8*

#### Theme 2: Medication Return Program

All the 18 respondents mentioned that medication return services in their pharmacies were low and rare. They perceived that the lack of demand for such services from their customers and lack of public awareness were the main reasons for the absence of such services.

“*I agree that the medicine return program is important in promoting safe medicine disposal. As far as I know, there are no formal guidelines or requirements for community pharmacies to accept medicine returns from the public. It is also the responsibility of the chain pharmacies as well. We cannot always rely on the government to execute the schemes. The companies should come forward and run these kinds of schemes at least as their CSR (Corporate Social Responsibility) activity Hence, I don*'*t see the need for such programs in community pharmacies exist in the first place without any formal guidelines. It is better to have a guideline first for community pharmacies” R6*

*I personally had never received any request from my customers to return medicines, nor questions regarding how to dispose of medicines correctly.” R18*

*I think, perhaps it*'*s due to the low public awareness on safe medicine disposal practices.” R3*

### Domain 2: Availability of Medicine Return Service in Community Pharmacies

All the 18 respondents agreed that a medicine return service should be available in community pharmacies.

#### Theme 3: Driving Factors for Medicines Return Service

Fourteen respondents considered that they were ready to provide medicines return services at their pharmacies. Respondents mentioned five key driving factors.

##### Sub Theme 3.1: Environmental Impacts and Risk of Child Poisoning

Ten respondents considered environmental impact and the potential risks of unsafe medicine disposal as the reasons for their readiness to provide medicines return services. Respondents spoke of the risks that children might ingest unwanted medicines.

“*If medicine return service is not available for the people, they will start dumping medicines in backyards” R4*

“*There are chances the medicine wastage becomes unsafe for the kids at home and it may affect the safety environment at home as well. Cases have been reported about children using expired or unused medicines either intentionally or unintentionally” R15*

##### Sub Theme 3.2: Convenient Location

Twelve respondents mentioned that providing a convenient location for consumers to dispose of unwanted medicines would encourage their return. Respondents said that collaboration with non-profit organizations may help drive medicines return programs.

“*I think there should be a system to collect the unused medications from the residential areas. Pharmacies are one such place” R8*

*The location to return the medicines should be conveniently reachable by the public. Hence unused medicine collection points can be initiated along with the NGOs (Non-Governmental Organization)” R11*

##### Sub Theme 3.3: Promote Government Initiative

Eight respondents wanted to stand by the government initiatives as an act of support to encourage the practices of safe medicine disposal. They feel that it is their responsibility to take forwards the government initiatives. This shows that pharmacists are key to promoting government initiatives, though there are no clear guidelines for safe medicine disposal.

“*I think as a pharmacist I should stand by the government schemes and programs that promote health and well-being” R2*

“*I feel it*'*s our responsibility to involve in government schemes and encourage the public to use those schemes” R11*

##### Sub Theme 3.4: Support From the Company

Ten respondents mentioned that any medicines return service should be enforced and supported at the company level. They also mentioned that it should be seen as the companies' corporate social responsibility.

“*It is the responsibility of the chain pharmacies as well. We cannot always rely on the government to execute the schemes. The companies should come forward and run these kinds of schemes at least as their CSR (Corporate Social Responsibility) activity” R6*

##### Sub Theme 3.5: Incentives From the Government

Nine respondents wanted to receive incentives from the government for providing such a service.

“*To consider providing medicine return service at the retail pharmacy, there should be some sort of help like incentives from the government, as the process can be an extra workload for the pharmacy staff and an increase in operational cost for the pharmacy.” R10, R5*

#### Theme 4: Deterring Factors Toward Medicine Return Service

The reasons that the 18 respondents perceived for the lack of medicines return services in most community pharmacies are:

##### Sub Theme 4.1: Lack of Guidelines

Fifteen respondents mentioned that there should be definite guidelines for community pharmacies in terms of medicine return programs.

“*As far as I know, there is no formal guidelines or requirements for community pharmacies to accept medicine returns from the public. It is better to have a guideline first for community pharmacies” R6*

##### Sub Theme 4.2: Operational Costs and Incineration Fees

Nine respondents mentioned that charges for the incineration of returned medications are determined by the weight, thus collecting medications from the public in large amounts would be unfavorable to the company's operational costs, unless there are subsidies from the government and consumers are willing to bear the extra costs of the medicine return service.

“*Accepting medicine returns from the public is unfavorable, as the waste management company charges the medicine waste incineration fees based on weight. The more waste there is, the more expensive the incineration fees would be.” R5*

### Domain 3: Management of Unwanted, Expired, and Returned Medicines

#### Theme 5: Close-Expiry Medicines

Thirteen respondents cleared close-expiry medication stocks in their pharmacy by selling them at lower prices to consumers and private clinics, or by transferring them to other pharmacies with higher stock movement. Most of the respondents mentioned that expired medication stocks would mean a profit loss to the pharmacy and hence the number of expired stocks for any effectively managed pharmacies would be kept at a minimal level.

“*Any medication waste in a community pharmacy meant revenue loss.” R13*

“*We will try to sell the close-expiry stocks out at discounted prices to customers or transfer them to other outlets with higher stock movement to avoid profit loss.” R4*

“*We will order according to demands and stock movements, and try to minimize inventory of slow-moving products”R17*

#### Theme 6: Expired Medicine Stocks and Medicinal Wastage

Eleven respondents mentioned that the amount and type of the expired medications would be documented before transferring the expired medications to the headquarters or warehouse for centralized disposal and incineration by qualified waste management companies.

“*For expired medications, we will record the name, type and amount of the expired stocks before sending them to the company warehouse for incineration. This is to ensure that no products are lost due to theft and for documentation purposes.”R1*

“*If the supplier or seller of the medication accepts returns of expired stocks, we will return the stocks to them in exchange of new stocks or rebates.”R9,R18*

#### Theme 7: Medicine Returns From the Public

Sixteen respondents did not accept medicine returns from customers at their pharmacies. Some respondents suggested that customers utilize the Medication Return Program (MRP) available at government healthcare facilities, while some did not suggest any alternative safe medicine disposal methods to their consumers.

“*We do have a pharmacy outlet in Bangsar that accepts medicine returns from the public. In my outlet, we would accept the returned medications and transfer them to that outlet for safe disposal.”R1*

“*I would just tell the customers that we do not accept medicine returns. I will not tell them how to dispose of the medicines proactively unless they asked, which I would recommend them to go to any government healthcare facilities for proper disposal.”R8*

### Domain 4: Minimization of Medicinal Wastage

#### Theme 8: Pharmacy Stocks

To reduce the possibility of ending up with expired medication stocks, 10 respondents reported practicing First-Expiry-First-Out (FEFO) while selling and arranging the stocks on the shelves. Periodic stock checks to check the expiry dates for different products are also conducted to minimize the amount of close-expiry medicine stocks. For expensive and rarely used medicines, pharmacists will only order the item as needed.

“*When we receive new stocks, we will arrange the stocks according to the expiry dates so that the old stocks could be sold first.”R8*

“*We perform checking of product expiry dates weekly to keep a record of the stock shelf-life and to decide which product to preferably sell first.”R2*

#### Theme 9: Dispensed Medications

To minimize the bulk-storing of medications by customers, which may lead to wastage, 12 respondents mentioned that they advise customers to frequently check the medications' expiry dates when making a purchase or for medications stored at home.

“*We do ask the customers to check the expiry dates of medications which are there at their homes and advise them not to store too many medications of the same kind.”R15*

“*We recommend customers to keep their medicines at the cool dry area in their house, and to avoid places like the car or washroom, which are not the ideal environment for medicine storage.”R3*

## Discussion

In this study, we explored the opinions of community pharmacists in the management of medication wastage, medicine return, and safe medicine disposal in Malaysia; we sought their views on the challenges involved and the feasibility of medicine return and safe disposal in a community pharmacy setting.

Respondents perceived that safe medicines disposal and medication return programs would be beneficial for their customers and the public. The main reason contributing to medicines wastage is non-adherence and poor adherence to medicine return program (4–6). The study also revealed that customers purchasing medications from community pharmacies are less likely to end up with leftover medications, because the customers had to pay out of their own pockets, unlike in most government healthcare facilities where medications are dispensed free, or at very low cost. The participants identified no specific approaches to their management of medicine returns from the public, and the methods differed from one outlet to another. Availability of the medicine return services in a pharmacy is based on whether the nearby healthcare facilities offer MRP. This concurs with a study by Smale et al. (13). Hence, in most pharmacies, pharmacists do not accept medicines returned from the public. Some pharmacists voluntarily collected the returned medicines and sent them to nearby healthcare facilities that offer MRP or to those pharmacy outlets, especially chain pharmacies, which provide MRP services. None of the returned medications are re-dispensed, resold, or donated to non-profit organizations.

Participating community pharmacists wanted the medicine returns service to be available in their pharmacies and mentioned environmental impact and the risk of child poisoning that can occur with unused and expired medicines. A previous study in Malaysia revealed that pharmaceutical substances were the causative agents in 40.5% of cases of intentional poisoning, and 30% of cases of unintentional poisoning; 46% of the victims of unintentional poisoning cases were children aged between 1 to 9 years (14). Another study stated that the largest contributing factor in pediatric poisoning was pharmaceutical substances, accounting for 39.2 % of the reported cases (15).

Respondents mentioned that a convenient location for the public to return their medicines would be a driving factor for people to return medicines. Different countries employ various strategies to manage expired and unwanted medicine returns from the public. In the United States, the Drug Enforcement Administration (DEA) conducts National Prescription Drug Take-Back Day twice annually, where participating local law enforcement agencies, retail pharmacies, hospitals or clinics from communities nationwide set up collection points for unwanted medications for safe disposal (16). In addition, there are all-year-round medicine disposal programs and DEA-authorized collectors in many local communities that facilitate the public in the safe disposal of unwanted medications (17). Moreover, there are many DEA-authorized collection bins located in many local community pharmacies in the United States. However, in Malaysia, there are no such collection points or government authorized collectors. In Malaysia, the MRP program is known as “Return Your Medicines”, which was introduced by the Pharmaceutical Services Division of the Ministry of Health Malaysia (MOH) in 2010 and allows the public to return unused medications to pharmacy counters in any government health facility for safe disposal (10); however, there is no compulsory participation in MRP for private-owned healthcare facilities and pharmacies. The present findings suggest that pharmacists would support government-led healthcare initiatives, provided they receive incentives and support either from the government or from their organizations. The study indicates that Malaysian community pharmacists are ready to take on this task with reasonable remuneration for the service. Factors that deter pharmacists from using the medicine returns service include lack of guidelines by the government and operational costs along with incineration fees. From a health economics standpoint, medicines wastage is wastage of budgetary resources and an opportunity cost. Studies in Malaysian public hospitals have shown that average monthly medicines wastage was much higher than that could have been spent per patient treatment (18, 19). Hence, operational costs for safe disposal remain a quandary during the budget allocation for any government. Though Malaysia does not have specific guidelines for medicines return, a legislative framework governs the disposal of hazardous and scheduled wastes from healthcare facilities and manufacturing premises (20). The Environmental Quality (Scheduled Wastes) Regulations 2005 (EQSWR) applied the cradle-to-grave approach in its management principles of scheduled waste; this means that the onus is on the waste generators to manage the hazardous waste from the creation until its ultimate disposal, for example, by appointing a licensed contractor to collect the waste for disposal at scheduled incinerators or secure landfills. EQSWR regulates the generation, storage, treatment, transport, and disposal of scheduled waste, including unwanted and expired pharmaceuticals, only by industrial premises, hospitals and other healthcare institutions and facilities (20).

The management of unwanted expired and returned medicines could be achieved by effective stock control of close-expiry and expired medicine. Pharmacists tend to minimize the retention of expired medicines as this would affect the pharmacy's profit. Instead, close-expiry medications are sold at a lower price to cover costs and minimize profit loss. Expired medications are usually collected at a centralized company facility, which then is managed and incinerated by external waste management companies. There are other methods of reducing medicine wastage such as exchanging stocks with other pharmacies, medication reconciliation with customers and adjusting the quantities during dispensing, especially during the treatment initiation stage where patients are more likely to discontinue their treatment (21–24). Re-dispensing of returned medications is not practiced in Malaysia because of concerns regarding risks to patients arising from the condition of returned medications and the presence of counterfeit products (25). However, many studies support the re-use of returned unexpired medicines as a cost-effective method of minimizing medicines wastage (3).

Studies have suggested that minimizing medical wastage improves the quality of the healthcare system through better utilization of healthcare resources, allowing healthcare professionals to emphasize patient care (26–28).

## Recommendations

In Malaysia, there is no specific directive on how members of the public should manage unwanted medicines. Doctors and pharmacists could minimize medicines wastage by avoiding overprescribing and encouraging consumers to return unwanted and expired medicines for safe disposal. Further studies are required on consumers' attitudes regarding the return of medicines and their safe disposal.

## Limitations

This study has some limitations. In this study, purposive and snowball sampling techniques were used. Though these methods helped the researchers to discover the study characteristics of this population, sampling bias might have affected the study. Also, the sampling technique could have led to the chances of selection bias. This study focused on specific issues in a particular set of populations, hence caution should be made while generalizing the research findings. In this study, the respondents are from chain pharmacies. Hence the results may not depict the scenarios of independent pharmacies.

## Conclusions

This qualitative study revealed the perception of community pharmacists regarding the management of medicine wastage, medicine return, and safe disposal of medicines in Malaysia. Medication wastage due to unused medications in community pharmacies was kept minimal to reduce profit loss. Medicine returns were normally not accepted in community pharmacies due to operational costs except for a handful of pharmacies outlets that had the Medicines Return Program (MRP) supported at their organizational level. Disposal of unused medications was undertaken by centralizing the stocks at an organization facility before being disposed of by outsourced waste management companies. Pharmacists mentioned that MRP in community pharmacies would be widely used if there was increased public awareness about safe medicine disposal. Moreover, there is an increased need for such a service; implementation of the official MRP in community pharmacies would require government subsidies.

## Data Availability Statement

The original contributions presented in the study are included in the article/supplementary material, further inquiries can be directed to the corresponding authors.

## Ethics Statement

The studies involving human participants were reviewed and approved by International Medical University (IMU) Joint-Committee on Research and Ethics. The patients/participants provided their written informed consent to participate in this study.

## Author Contributions

KR and KC: conceptualization and data curation. KR and MM: methodology, writing—review and editing, project administration, and funding acquisition. DC: validation, resources, and visualization. KC: formal analysis, investigation, and writing—original draft preparation. KR, DC, and MM: supervision. All authors contributed to the article and approved the submitted version.

## Funding

This research was funded by the Institute for Research, Development and Innovation (IRDI), International Medical University, Malaysia (MPP I-2020(07)).

## Conflict of Interest

The authors declare that the research was conducted in the absence of any commercial or financial relationships that could be construed as a potential conflict of interest.

## Publisher's Note

All claims expressed in this article are solely those of the authors and do not necessarily represent those of their affiliated organizations, or those of the publisher, the editors and the reviewers. Any product that may be evaluated in this article, or claim that may be made by its manufacturer, is not guaranteed or endorsed by the publisher.
